# Age-specific acceleration in malignant melanoma

**DOI:** 10.12688/f1000research.10491.2

**Published:** 2017-02-24

**Authors:** Brian L Diffey, Steven A Frank

**Affiliations:** 1 Dermatological Sciences, Institute of Cellular Medicine, Newcastle University, Newcastle upon Tyne, UK; 2 Department of Ecology and Evolutionary Biology, University of California, Irvine, USA

**Keywords:** melanoma epidemiology, age-period-cohort effects, sun exposure, age-specific incidence

## Abstract

**Background:** The overall incidence of melanoma has increased steadily for several years. The relative change in incidence at different ages has not been fully described. **Objective:** To describe how incidence at different ages has changed over time and to consider what aspects of tumour biology may explain the observed pattern of change in incidence. **Methods:** The slope of incidence *vs* age measures the acceleration of cancer incidence with age. We described the pattern of change over time in the overall incidence of melanoma, as well as in acceleration. We used data for males and females from 3 different countries in the 17 sequential 5-year birth-cohort categories from 1895-99 to 1975-79, from which we derived the incidence patterns. **Results:** Over time, there has been a tendency for the overall incidence of melanoma to increase and for the acceleration (slope) of the age-incidence curves to decline. The changing patterns of melanoma incidence and acceleration differ between males and females and between the countries analysed. **Conclusions:** The observed pattern in melanoma of rising incidence and declining acceleration occurs in other cancers in response to genetic knockouts of mechanisms that protect against cancer. Perhaps some protective mechanism with respect to melanoma may be less effective now than in the past, possibly because of more intense environmental challenges.

## Introduction

The incidence of malignant melanoma has increased steadily over the past 50 years in predominately fair-skinned populations ^1^. The trends in incidence probably reflect changing prevalence of risk factors such as increased leisure time in sunny destinations, changing fashion and sunbed use, coupled with increased surveillance, early detection and changes in diagnostic criteria ^2, 3^.

The purpose of this paper is to study the particular ways in which incidence has changed over time. By analysing the 17 sequential 5-year birth cohorts from 1895–99 to 1975–79, we show that incidence has indeed increased steadily over time. Our analysis also shows that the particular patterns of increase in incidence differ between males and females and between different countries.

In addition to the overall increase in incidence, the relationship between age and incidence has also changed over time. We show that more recent cohorts typically have a disproportionate increase in cases at earlier ages.

To quantify the age-incidence relationship and its change over time, we study the rate of change of melanoma incidence with age ^4– 6^, which is the acceleration of cancer ^7^. The patterns of acceleration provide interesting information about the forces acting on cancer progression at different ages ^8^.

## Methods

Age-specific incidence data on malignant melanoma (ICD-10; C43) for males and females were obtained for Great Britain ^9– 11^ for the period 1975–2014, the USA ^12^ for the period 1975–2013 and Australia ^13^ for the period 1982–2012. Incidence data for the USA relate to white people only.

Because the incidence of melanoma is increasing over time, age-specific rates are heavily influenced by the year of birth. To allow for this effect, we separated the 17 sequential 5-year birth-cohort categories from 1895–99 to 1975–79. For each cohort, we computed the 5-year average age-specific incidences for males and females aged 25 years and over.

The analyses were done with Microsoft Excel 2003.

## Results

**Figure 1.**
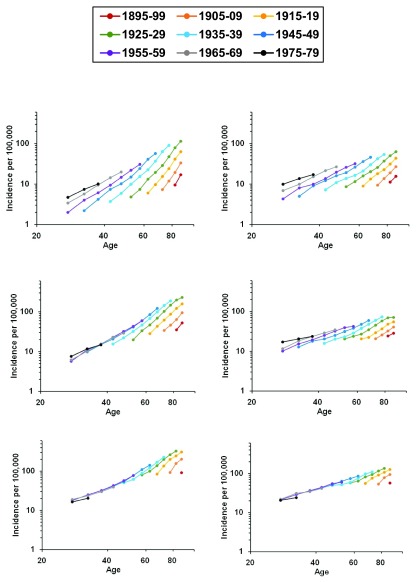
The age-specific incidence of melanoma in different time periods and different countries. The plots show the incidence for males (left) and females (right) in Great Britain (top row), USA (middle row), and Australia (bottom row) for the birth cohorts shown in the top legend. The plots do not show the intermediate decadal cohorts because of visual limitations in plotting the data. The plots are based on the summary given in Dataset 1, derived from the data and analyses in Dataset 2– Dataset 5. Both axes are scaled logarithmically.

Table 1 shows the age-specific incidence for British males born during different time periods. The risk of malignant melanoma within each cohort rises consistently throughout life, as is true for most other cancers ^8^. Figure 1 shows the age-incidence curves for both genders from Great Britain, the USA, and Australia for successive birth cohorts from 1895–99 to 1975–79.

**Table 1.** The age-specific incidence per 100,000 man-years of malignant melanoma in British males averaged in 5-year intervals.

	Age Specific Rate (5-y average) in Birth Cohort																
Age Band	1895–99	1900–04	1905–09	1910–14	1915–19	1920–24	1925–29	1930–34	1935–39	1940–44	1945–49	1950–54	1955–59	1960–64	1965–69	1970–74	1975–79
25–29												1.5	2.0	2.9	3.4	4.1	4.7
30–34											2.2	2.9	4.0	4.7	5.7	6.8	7.4
35–39										3.2	4.2	5.4	6.1	7.2	9.5	10.5	10.1
40–44									3.7	5.2	7.4	7.7	9.4	12.4	14.4	14.2	
45–49								4.2	5.9	8.5	10.2	11.8	14.7	17.0	20.0		
50–54							4.8	7.0	9.9	12.5	14.9	18.1	22.0	24.2			
55–59						5.0	7.4	11.4	15.4	17.9	24.3	28.7	30.8				
60–64					6.0	8.9	13.2	16.9	22.6	31.3	41.1	44.1					
65–69				6.5	9.7	14.2	19.5	26.1	36.8	50.7	57.1						
70–74			7.3	10.4	15.4	20.9	28.8	44.3	63.6	73.8							
75–79		8.9	11.9	18.5	24.1	33.6	47.5	71.4	90.8								
80–84	9.5	13.5	19.4	29.1	41.6	56.3	79.4	103.5									
85+	17.0	24.6	33.3	43.9	63.2	90.1	114.9										

From Figure 1, it appears that, over time, there has been a tendency for the acceleration (slope) of the incidence curves to decline. The decline in acceleration over time seems particularly strong for certain datasets shown in Figure 1, for example, for British males. Other datasets, such as Australian females, seem not to show a clear trend. Thus, it is helpful to make a more direct analysis for the changing acceleration patterns between the different datasets.

**Figure 2.**
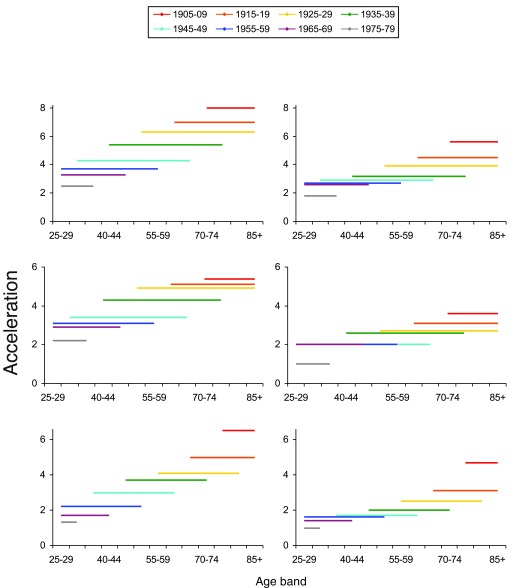
The age-specific acceleration of melanoma in different time periods and different countries. The plots show the acceleration for males (left) and females (right) in Great Britain (top row), USA (middle row), and Australia (bottom row) for the birth cohorts shown in the top legend. The plots do not show the intermediate decadal cohorts because of visual limitations in plotting the data. The plots are based on the summary given in Dataset 6, derived from the data and analyses in Dataset 2– Dataset 5.

To describe the tendency for age-specific acceleration to decline over birth cohorts, we calculated the following summary statistics separately for each of the 6 datasets represented by the 6 panels in Figure 2. In each successive pair of the 17 cohorts, we used data only for the common ages shared by the two cohorts. For those common ages, we estimated by linear regression the slope of the log-log age-incidence data, which estimates the age-specific acceleration. We then calculated the ratio of the accelerations for the more recent cohort relative to the prior cohort, and used the logarithm base 2 value of that ratio. A negative value means the more recent cohort has a lower slope.

The average of the logarithms over the successive pairs of cohorts describes the geometric mean of the slopes, capturing the multiplicative tendency of the slope to change over cohorts. A negative value expresses an overall tendency for the slope to decline over time.

To gain a sense of the trend in acceleration over the successive cohorts, Table 2 shows, for each of the 6 datasets, the average logarithm for the ratio of successive slopes, and the standard error of that average. We also calculated the average logarithm divided by the standard error of that average, which gives the deviation from zero in terms of the number of standard errors of the mean.

**Table 2.** Logarithm to base 2 of the ratio of slope in a given birth cohort to that in the adjacent older cohort determined only across those ages that both cohorts have in common.

Birth Cohort																				
	1900–04	1905–09	1910–14	1915–19	1920–24	1925–29	1930–34	1935–39	1940–44	1945–49	1950–54	1955–59	1960–64	1965–69	1970–74	1975–79		Mean	SE	Ratio mean:SE
**Males**																				
GB	0.03	0.01	-0.07	-0.03	-0.04	-0.05	-0.02	-0.02	-0.07	-0.06	-0.07	-0.09	-0.12	-0.02	-0.20	-0.28		-0.07	0.02	-3.54
USA	-0.19	-0.01	-0.04	-0.13	0.01	-0.04	-0.10	-0.01	-0.05	-0.08	-0.06	0.01	-0.03	-0.03	-0.18	-0.33		-0.08	0.02	-3.45
Australia		-1.39	-0.70	-0.29	-0.19	0.13	0.07	0.07	0.07	0.07	-0.03	-0.12	-0.07	-0.23	-0.01	-0.16		-0.19	0.10	-1.83
**Females**																				
GB	0.43	-0.14	-0.29	-0.09	-0.08	-0.06	-0.08	-0.03	-0.13	-0.10	-0.08	-0.09	-0.03	-0.07	-0.12	-0.57		-0.10	0.05	-2.00
USA	0.73	-0.54	-0.15	0.13	0.04	-0.16	0.03	-0.03	-0.06	-0.15	-0.02	0.00	0.01	-0.18	-0.26	-0.99		-0.10	0.09	-1.13
Australia		-0.28	-1.00	-0.45	-0.26	0.28	0.10	0.22	0.04	0.06	-0.13	-0.03	-0.19	-0.11	-0.09	-0.83		-0.18	0.09	-1.93

The overall trends suggest that acceleration has declined over time, consistent with the general visual pattern shown in Figure 2. However, Table 2 shows that there is significant variation in the trends between genders and countries, also apparent from Figure 1 and Figure 2.

In every case the overall tendency over the cohorts has been for incidence to increase and acceleration (slope) to decline.

Summary data for Figure 1, age-specific incidence of melanoma in different time periods and different countries Click here for additional data file. Copyright: © 2017 Diffey BL and Frank SA 2017 Data associated with the article are available under the terms of the Creative Commons Zero "No rights reserved" data waiver (CC0 1.0 Public domain dedication).

Raw age-specific incidence data for Australia for different age groups in different years Data obtained from Australian Institute of Health and Welfare (AIHW) 2016, Australian Cancer Incidence and Mortality (ACIM) books: Melanoma of the skin, Canberra: AIHW. Available at http://www.aihw.gov.au/acim-books. Click here for additional data file. Copyright: © 2017 Diffey BL and Frank SA 2017 Data associated with the article are available under the terms of the Creative Commons Zero "No rights reserved" data waiver (CC0 1.0 Public domain dedication).

Raw age-specific incidence data for Great Britain for different age groups in different years Data obtained from (1) Office for National Statistics, Cancer Registration Statistics, England, available at http://www.ons.gov.uk/peoplepopulationandcommunity/healthandsocialcare/conditionsanddiseases/datasets/cancerregistrationstatisticscancerregistrationstatisticsengland, (2) Welsh Cancer Intelligence and Surveillance Unit, Cancer in Wales, available at: http://www.wcisu.wales.nhs.uk/cancer-in-wales-1, and (3) Information and Statistics Division Scotland, Cancer Statistics, available at: http://www.isdscotland.org/Health-Topics/Cancer/Cancer-Statistics/Skin/
Copyright: © 2017 Diffey BL and Frank SA 2017 Data associated with the article are available under the terms of the Creative Commons Zero "No rights reserved" data waiver (CC0 1.0 Public domain dedication).

Raw age-specific incidence data for USA for different age groups in different years Data obtained from Surveillance Research Program of the Division of Cancer Control and Population Sciences, National Cancer Institute, available at: http://seer.cancer.gov/seerstat
Copyright: © 2017 Diffey BL and Frank SA 2017 Data associated with the article are available under the terms of the Creative Commons Zero "No rights reserved" data waiver (CC0 1.0 Public domain dedication).

Transformation of raw data in Datasets 2–4 into summary statistics used in the figures and analyses and in Table 1 and 2 Click here for additional data file. Copyright: © 2017 Diffey BL and Frank SA 2017 Data associated with the article are available under the terms of the Creative Commons Zero "No rights reserved" data waiver (CC0 1.0 Public domain dedication).

Summary data for Figure 2, age-specific acceleration of melanoma in different time periods and different countries Click here for additional data file. Copyright: © 2017 Diffey BL and Frank SA 2017 Data associated with the article are available under the terms of the Creative Commons Zero "No rights reserved" data waiver (CC0 1.0 Public domain dedication).

## Discussion

We analysed the incidence of malignant melanoma in 6 separate datasets representing males and females from Great Britain, the United States, and Australia, locations with large differences in ambient solar ultraviolet radiation, which is regarded as a major aetiological factor in the disease. Because the incidence of melanoma has tended to increase over time, we calculated the patterns of incidence separately for 17 successive 5-year birth cohorts between 1895 and 1979 in each of the 6 datasets.

In our analysis, we calculated the age-specific incidence separately for each cohort. We also calculated the acceleration of cancer incidence with age for each cohort, in which acceleration is the rate of increase in incidence with age described by the slope of the log incidence *vs* log age plots.

The tendency over the cohorts has been for incidence to increase and acceleration to decline over time. Figure 1 summarizes the incidence patterns, in which the higher position of the curves with the passing of time expresses the rise in incidence. In that figure, one can also see a tendency for the slope to decline with the passing of time, which corresponds to a decline in acceleration. Figure 2 and Table 2 provide a more detailed summary of the way in which acceleration has tended to decline with the passing of time. The variation between sexes and between countries is clear but unexplained.

It is evident that observed incidence data on melanoma over time are subject to the influence of many factors that include period effects and cohort effects.

Period effects can be regarded as resulting from external factors that affect equally all age groups at a particular calendar time and could be a consequence of economic, environmental or social factors; for example, depletion of the ozone layer resulting in higher levels of ambient ultraviolet radiation. Also, methodological changes in outcome definitions, classifications, or method of data collection, such as increased surveillance, early detection and changes in diagnostic criteria, could also lead to period effects in data.

Cohort effects, on the other hand, result from the unique experience/exposure of a particular group, or cohort, of subjects as they move across time leading to differences in the risk of outcome based on birth year. For example, following the widespread introduction of sunbeds for cosmetic tanning in the 1980s and their popularity amongst younger people, it would be expected that cohorts born after 1960 would be greater users of this form of UV exposure than cohorts born in earlier years. Some factors may influence periods or cohorts depending on their effects, such as educational awareness and prevention campaigns that act at particular times (periods) but may be targeted to certain age groups (cohorts).

The most interesting trend in the data concerns the tendency over time for incidence to rise and acceleration to decline ( Figure 1 and Figure 2). In other cancers, various genetic and environmental perturbations cause a similar pattern of rising incidence and declining acceleration ^8, 14– 16^. In some of those cases, the rise in incidence and decline in acceleration appears to follow from abrogation of one or more of the normal genetic or physiological restraining processes that protect against cancer.

In the patterns of melanoma incidence that we observed, it is not clear if the observed rise in incidence and decline in acceleration over time arose from a similar abrogation of a normally restraining genetic or physiological process, perhaps as a consequence of changes in behaviour, education, diagnosis, environment or other common modulators of observed cancer incidence. As further understanding of environmental, genetic and physiological causes of melanoma develops, it will be interesting to determine how changes in particular causes relate to the changing patterns of incidence over time.

## Data availability

The data referenced by this article are under copyright with the following copyright statement: Copyright: © 2017 Diffey BL and Frank SA

Data associated with the article are available under the terms of the Creative Commons Zero "No rights reserved" data waiver (CC0 1.0 Public domain dedication). 

**Dataset 1. Summary data for Figure 1, age-specific incidence of melanoma in different time periods and different countries**. doi, 10.5256/f1000research.10491.d148748 ^17^

**Dataset 2. Raw age-specific incidence data for Australia for different age groups in different years**. Data obtained from Australian Institute of Health and Welfare (AIHW) 2016, Australian Cancer Incidence and Mortality (ACIM) books: Melanoma of the skin, Canberra: AIHW. Available at http://www.aihw.gov.au/acim-books. doi, 10.5256/f1000research.10491.d148749 ^18^

**Dataset 3. Raw age-specific incidence data for Great Britain for different age groups in different years**. Data obtained from (1) Office for National Statistics, Cancer Registration Statistics, England, available at http://www.ons.gov.uk/peoplepopulationandcommunity/healthandsocialcare/conditionsanddiseases/datasets/cancerregistrationstatisticscancerregistrationstatisticsengland, (2) Welsh Cancer Intelligence and Surveillance Unit, Cancer in Wales, available at: http://www.wcisu.wales.nhs.uk/cancer-in-wales-1, and (3) Information and Statistics Division Scotland, Cancer Statistics, available at: http://www.isdscotland.org/Health-Topics/Cancer/Cancer-Statistics/Skin/. doi, 10.5256/f1000research.10491.d148750 ^19^

**Dataset 4. Raw age-specific incidence data for USA for different age groups in different years.** Data obtained from Surveillance Research Program of the Division of Cancer Control and Population Sciences, National Cancer Institute, available at: http://seer.cancer.gov/seerstat. doi, 10.5256/f1000research.10491.d148751 ^20^

**Dataset 5. Transformation of raw data in Dataset 2– Dataset 4 into summary statistics used in the figures and analyses and in Table 1 and Table 2**. doi, 10.5256/f1000research.10491.d148752 ^21^

**Dataset 6. Summary data for Figure 2, age-specific acceleration of melanoma in different time periods and different countries**. doi, 10.5256/f1000research.10491.d148753 ^22^
