# Supplementary material for: Age-specific acceleration in malignant melanoma
Source: F1000Res. 2017 Feb 24;6:27. Originally published 2017 Jan 9. [Version 2] doi: 10.12688/f1000research.10491.2 (PMC5381631; doi:10.12688/f1000research.10491.2)
Supplement: Peer review history

# Peer review: Referee response for version 2

**DOI:** 10.5256/f1000research.11884.r21403

**Published:** 2017-03-30

**Authors:** Antony Young

**Affiliations:** 1 St John’s Institute of Dermatology, King's College London, London, UK

I am happy with the revision and hope the paper stimulates research into possible reasons/mechanisms for the observations.

I have read this submission. I believe that I have an appropriate level of expertise to confirm that it is of an acceptable scientific standard.

# Peer review: Referee response for version 2

**DOI:** 10.5256/f1000research.11884.r20505

**Published:** 2017-02-24

**Authors:** Robert J. Noble

**Affiliations:** 1 Department of Biosystems Science and Engineering, ETH Zurich, Basel, Switzerland

The authors have addressed concerns raised in my review of the previous version, and I have no further comments.

I have read this submission. I believe that I have an appropriate level of expertise to confirm that it is of an acceptable scientific standard.

# Peer review: Referee response for version 1

**DOI:** 10.5256/f1000research.11306.r19972

**Published:** 2017-02-17

**Authors:** Robert J. Noble

**Affiliations:** 1 Department of Biosystems Science and Engineering, ETH Zurich, Basel, Switzerland

This well presented study describes an interesting trend in melanoma incidence in three industrialised nations and proposes an explanatory hypothesis. The major caveat is that, as the authors acknowledge, this trend could be due to any number of cohort effects (i.e. changing environmental and social factors that unequally affect different age groups and cohorts). The decrease in acceleration of melanoma incidence could, for example, be due to changes in skin cancer prevention campaigns, foreign travel, fashion, sunbathing behaviour, sunbed use, or frequency of dermatological examination. As the authors do not control for any such factors and provide no evidence for or against any particular hypothesis, they can do no more than speculate about how the observed pattern arose.

 Also importantly, it is unclear to me exactly what the authors mean by a “restraining process” or “protective mechanism” that might have become less effective over time, nor how such a process might be affected by “a change in environmental exposure patterns” or “more intense environmental challenges”. I suspect they mean to suggest that a germline mutation that inactivates a tumour suppressor gene has become more prevalent due to natural selection or genetic drift, but I am unsure if this interpretation is correct. In any case, it would be useful to have more details of the hypothesis and its testable predictions. Would the authors expect to see a similar trend in other cancer types? Why might some countries be more affected than others? And why might men and women be affected differently?

 The study might also benefit from using a mathematical model (as, for example, in reference 14) to estimate how much decrease in prevalence of the protective mechanism would be necessary to explain the observed trends.

I have read this submission. I believe that I have an appropriate level of expertise to confirm that it is of an acceptable scientific standard, however I have significant reservations, as outlined above.

# Response

**Published:** 2017-02-21

**Authors:** Steven Frank

**Affiliations:** Department of Ecology & Evolutionary Biology, University of California, Irvine, USA

We thank Robert Noble for his comments and helpful criticisms.

 The main comment in Robert Noble’s review suggested that we add more detail about our mechanistic hypothesis. The other referee, Antony Young, made the opposite comment, suggesting that we delete our discussion of mechanism because it is too speculative.

 After considering these opposing suggestions, we decided to delete our previous discussion of possible mechanisms that could explain the observed rise in incidence and decline in acceleration. The entire focus of our analysis and presentation concerned the patterns of incidence over time in the available data. We have no profound insight into the possible mechanistic causes of the interesting patterns that we observed. Thus, we have chosen in the revision to keep the focus on the data and the patterns that emerged from our analysis.

 We remain very interested in the puzzle that has emerged from our analysis. However, we now think it best to defer any mechanistic discussions and model development until we have a more compelling argument that could be published as a follow up study to the data in this article.

# Peer review: Referee response for version 1

**DOI:** 10.5256/f1000research.11306.r20144

**Published:** 2017-02-13

**Authors:** Antony Young

**Affiliations:** 1 St John’s Institute of Dermatology, King's College London, London, UK

I found this paper very interesting but hard to follow at times. The 2 ^nd^ column of the discussion seems to be at bit contradictory. The authors give possible mechanistic explanations for their observations then draw attention to the many uncertainties in the final paragraph. My own view is that these uncertainties are so large that it is too speculative to comment on biological mechanisms. The sex and country differences are apparent in the figures but it would be interesting to comment on these in the discussion. Can anything be explained by aggressive public health campaigns in Australia?

 Specific points

1. What is the scaling of the x axis of Figure 1?
2. Different colour codes are used in Figures 1 and 2 which I found confusing
3. Are educational and prevention campaigns period effects? I would have thought that they are cohort effects, because the age group that they affect is likely to be important
4. Should sunscreens be mentioned? Their possible role in melanoma has been analyzed in several studies
5. Can anything be deduced about latitude effects?

I have read this submission. I believe that I have an appropriate level of expertise to confirm that it is of an acceptable scientific standard, however I have significant reservations, as outlined above.

# Response

**Published:** 2017-02-21

**Authors:** Steven Frank

**Affiliations:** Department of Ecology & Evolutionary Biology, University of California, Irvine, USA

We thank Antony Young for his comments and helpful criticisms.

 In response to the criticism that our discussion of mechanism raised too many uncertainties, we have deleted those paragraphs. In the revision, we replace that part of the discussion with a brief summary of the key result: the observed rise in incidence and decline in acceleration over time. We then add a couple of sentences about the range of factors that may be involved and how those factors may act via the normal genetic and physiological processes that protect against cancer, by analogy with other cancers for which there is more genetic and physiological information about the relation between mechanism and patterns of incidence.

 With regard to specific comments:

 We added a description of the scaling of axes to the legend of Figure 1.

 We clarified that education and prevention campaigns could act as either period or cohort effects, depending on the targeting of age groups.

 The suggestions about public health campaigns in Australia, sunscreens, and latitude are all interesting possibilities. We do not have sufficient data or insight to say anything compelling about those issues, so we have not added any new analyses or discussion to the revision.
